# Building Implementation Science in Nutrition

**DOI:** 10.1093/advances/nmaa066

**Published:** 2020-06-25

**Authors:** Andrea M Warren, Edward A Frongillo, Rahul Rawat

**Affiliations:** Department of Health Promotion, Education, and Behavior, University of South Carolina, Columbia, SC, USA; Department of Health Promotion, Education, and Behavior, University of South Carolina, Columbia, SC, USA; Bill & Melinda Gates Foundation, Seattle, WA, USA

**Keywords:** implementation, science, nutrition, evaluation, interventions

## Abstract

The field of nutrition has been investing in the development of many nutrition-specific and -sensitive policies and programs aimed at improving population-level malnutrition in all its forms. When there is a need to learn about a new system, programmatic context, or target population to understand how to effectively deploy an intervention to help improve nutrition, it is important to be able to ask a broad range of questions, both in topic and in scope. Our aim is to provide a simple and conceptually clear definition and principles to elaborate the science of implementation for nutrition to distinguish it from other ways of knowing and learning and to serve as a guide to the articulation of implementation science questions and methods. Implementation science is a body of systematized knowledge about how to improve implementation that *1*) is distinguished by its aims to learn about the process of implementation, *2*) uses methods that derive from and fit with the aims, and *3*) is built with tacit (as well as expert) knowledge and experiential learning. Implementation science aims to generate the learning needed to improve implementation through facilitating collaboration among stakeholders to articulate and pursue the aims; capturing and using tacit knowledge and experiential learning from stakeholders, systems, providers, and recipients; and applying a mix of methods suited to the aims. This elaboration of the science provides a simple way to help those who already do, or want to do, implementation science understand and communicate how this science is unique and the value that it adds to the current landscape of nutrition priorities, innovations, and the attendant complex learning needs that follow. Implementation science encompasses both discovery- and mission-oriented research, and centers implementation as the object of study for the purposes of broad-based learning.

## Introduction

The field of nutrition encompasses an increasingly complex landscape of policies and interventions that target the immediate, underlying, and basic determinants of malnutrition, which builds on decades of research that positions nutrition as both a “maker and marker of development” (1–3). International and country-level nutrition strategies invoke systems-level thinking and increasingly engage multiple sectors simultaneously, particularly health, agriculture, education, and trade (4, 5). Achieving improvements in the nutrition of populations requires knowledge about biology and epidemiology, but the increasing complexity and multisectorality of nutrition initiatives highlight sociopolitical factors that determine which actions are appropriate and acceptable. With this, there is a growing need for knowledge that integrates across socioecological domains to determine how best to design and implement intended activities to achieve desired changes (6, 7). When there is a need to learn about a new system, programmatic context, or target population to understand how to effectively implement an intervention to help improve nutrition, it is important to be able to ask a broad range of questions, both in topic and in scope.

The field of nutrition has a long history, from its beginning >100 y ago, in building biological and epidemiological knowledge, and a much shorter history, roughly over the past 25 y, of building knowledge about nutrition policy (8). Concerted effort to build knowledge about implementation of nutrition policies and programs has a short history also, but its importance has been recognized particularly over the past 2 decades (6). The 2003 Lancet Child Survival Series, for example, identified 35 proven preventative and curative maternal and child health and nutrition interventions but noted that implementation with high coverage would be required to improve child survival at a population level (9–13). A subsequent review for the WHO of evidence on delivery strategies (i.e., how) and delivery points (i.e., where) found that there was limited evidence overall, with more evidence for some delivery points based in communities (i.e., home visits) or facilities (i.e., primary clinics, hospital inpatient care) than for others (i.e., community groups and assemblies, mobile clinics, immunization clinics, outpatient care) or for other delivery points (e.g., campaigns, mass media, schools). Only 25 of 35 interventions had ≥3 studies with evidence for ≥1 delivery strategy, and it was not clear how much evidence is enough for a given combination of intervention and delivery strategy (14).

For another example, evidence on effective interventions in nutrition in general suggests that they can be effective at low cost, but these studies come primarily from controlled environments, in short-term studies, or on small scales, whereas intervention implementation is needed in uncontrolled environments, at large scale (15). Recent efforts to improve infant and young child feeding through behavioral interventions, such as the Alive & Thrive initiative in 3 countries, and the Integrated Strategy for Attention to Nutrition initiative in Mexico, have demonstrated how intervention scale-up can be done quickly rather than waiting for accumulation of evidence over 15–20 y (15). These efforts involved bringing together practitioners and researchers outside and within countries with the intention, commitment, and planning to make a difference at large scale in a relatively short time from the outset (15–19).

Recent calls have been made for an organized body of knowledge and a community of practice around implementation science in nutrition that would enhance legitimacy for implementation science in nutrition, provide opportunities for implementation practitioners, and serve to increase recognition, rigor, and participation in this important effort (1, 20–22). This need is reflected in the establishment of the Society for Implementation Science in Nutrition (23), which has extensive material on its website and has sponsored a series of 9 well-attended webinars. Furthermore, the journal *Current Developments in Nutrition* has designated implementation science as a special topic of interest (24). Also, a total of 120 participants attended workshops that we convened on implementation science in nutrition at 2 international conferences in June 2019—the annual meeting of the American Society for Nutrition and the Agriculture, Nutrition, and Health Academy Week. These participants were enthusiastic about and experienced in conducting implementation studies in nutrition, and they provided important insights into the challenges facing the advancement of implementation science in nutrition.

In this article, first we build on recent work (20, 25) and a review of literature to describe 3 prominent perspectives on implementation science that apply to nutrition (and health). Second, we use the insights from the 2 workshops to discuss the state of implementation science in nutrition and the challenges that researchers and practitioners have identified. Third, we elaborate, in a simple and conceptually clear way, the science of implementation in order to distinguish it from other ways of knowing and learning and to serve as a guide to the articulation of implementation science questions and methods; we present a case study to illustrate the application of these ideas. Fourth, we discuss how to build implementation science in nutrition going forward.

## Three Prominent Perspectives in Implementation Science

Implementation science is not a new field of study (26, 27). The challenge highlighted by workshop participants of a lack of conceptual cohesion around definitions and methods in implementation science stems, in part, from lack of recognition of the distinct perspectives present within the broader field of implementation science as applied to public health. From our review of the current application of implementation science to nutrition, we identified 3 prominent perspectives from recent government and foundation funding opportunities and institutional literature: biomedical (28–30), program and policy (20, 31, 32), and health systems (33). These perspectives are different but not mutually exclusive, and each reflects the need to bridge research and practice. We compare and contrast them in terms of their basic characteristics, similarities, and dissimilarities to clarify the different concepts and their attendant methodologies (**Table 1**).

**TABLE 1:** Three primary perspectives on implementation science

	Biomedical	Policies or programs	Health systems
Starting point	The need to translate basic biomedical science into practice more quickly (largely oriented to clinical settings)	Improve implementation of a program or policy (de-emphasizes discovery-oriented science; primarily mission-oriented)	Understand systems’ functioning and alignment and articulation within and between different systems
Definition	“… the study of methods to promote the adoption and integration of evidence-based practices, interventions and policies into routine health care and public health settings” (29)	“an interdisciplinary body of theory, knowledge, frameworks, tools and approaches whose purpose is to strengthen implementation quality and impact” (20)	“… the scientific study of the processes used in the implementation of initiatives as well as the contextual factors that affect these processes” (33)
Purpose	Identify, understand, and develop strategies for overcoming barriers to the adoption, adaptation, integration, scale-up, and sustainability of evidence-based interventions, tools, policies, and guidelinesUnderstand when there is a need to “de-implement” interventions that are ineffective, unproven, low-value, or harmful	Identify and address implementation bottlenecksIdentify, evaluate, and scale up implementation innovationsEnhance the utilization of existing knowledge, tools, and frameworks based on the evolving science of implementation	Understand how health interventions “work in the real world”Capture and analyze information in real time to facilitate health systems strengtheningHelp organizations develop the capacity to learn from implementation—iterative process of knowledge generation and use from programming

The US NIH, which is a major funder of health science and sets standards for scientific rigor, is foundational to the biomedical perspective on implementation science and provides the most common definition in a recent funding announcement for dissemination and implementation research: “the scientific study of methods to promote the systematic uptake of proven clinical treatments, practices, organisational, and management interventions into routine practice, and hence to improve health.” Studies within the biomedical perspective might include the following types: “pilot or feasibility studies, secondary analysis of existing data, small, self-contained research projects, development of research methodology, and development of new research technology.” The funding announcement further stated: “The purpose of [this announcement] is to support innovative approaches to identifying, understanding, and developing strategies for overcoming barriers to the adoption, adaptation, integration, scale-up and sustainability of evidence-based interventions, tools, policies, and guidelines” (29). The “barriers and facilitators” framing frequently yields reductive, single-factor interpretations of a programmatic context and is not well suited to elucidating the complexities of implementation in real-world settings. (For further discussion on the importance of embracing contextual complexity in implementation research, see reference 34.) Furthermore, the focus on barriers to implementation and methods to overcome barriers represents a narrow facet of all possible implementation-related drivers and processes that are worthy of study. It also serves to focus attention to hypothesis-driven research to test methods to promote uptake against a specific barrier and de-emphasizes inductive and naturalistic forms of inquiry that would better serve to generate the understanding needed to improve implementation.

The programs-and-policies perspective is that implementation science is intended to address the gap in know-how for scaling up interventions to achieve, for example, the Sustainable Development Goals (20). A request for applications from the Eleanor Crook Foundation, for example, stated that “the request for applications is for implementation science projects designed to test innovations and delivery mechanisms (in terms of feasibility, acceptability, effectiveness, and/or efficiency) with the potential to increase the effectiveness of nutrition interventions and take them to scale.” This perspective emphasizes mission-oriented research and de-emphasizes discovery-oriented research: “Implementation research does not focus on research for academic purposes” (35). This distinction preserves the notion of a boundary between research and practice rather than bringing them together, and it does not acknowledge research initiatives that are undertaken as an integral part of program implementation (e.g., Suaahara in Nepal) and that both mission-specific and generalizable knowledge can be gained. With this perspective, a priority is placed on formative research to design a program or policy as well as research to identify implementation gaps of an existing program or policy and conduct studies to address the gaps (20, 31, 32).

The health-systems perspective is that “implementation research … addresses … the know–do gap in real-world settings” (25). This perspective defines implementation research as “… the scientific study of the processes used in the implementation of initiatives as well as the contextual factors that affect these processes” and promotes methods that “generate actionable intelligence, are good at capturing the subtleties of context over time, and offer the iterative flexibility needed to respond to change.” This perspective incorporates systems-level thinking that is helpful in understanding multisectoral landscapes. In this perspective, typical implementation science studies include pragmatic trials, effectiveness-implementation hybrid trials, quality improvement studies, and participatory action research (33).

Each of these 3 prominent perspectives about implementation science has arisen from needs recognized by the biomedical, program and policy, and health systems communities of researchers and practitioners. All 3 perspectives commonly view implementation science as a means of closing the gap between evidence and practice, but each community tends to see implementation science as producing research or compiling knowledge to provide information to address their respective needs. Not one of these perspectives captures the full range of what implementation science should be to function as a true science.

## State of Implementation Science in Nutrition

Participants of the 2 conference workshops in June 2019 pointed out that there is little agreement regarding definitions, methods, and curricula in implementation science. They also perceived a systemic undervaluing of implementation science, noting that governments and donors typically focus on end-line results and do not value achieving implementation as an outcome, thus limiting funding opportunities to carry out implementation science. Implementation science is infrequently built into programs and studies. Regarding broader communication within the community of practice, participants noted that implementation science is undervalued in academia and not widely shared and disseminated at academic conferences, and that it is challenging to reach and engage with nutrition practitioners.

Analysis of these issues points to an underlying lack of conceptual cohesion around implementation as a legitimate object of scientific inquiry. The lack of conceptual cohesion reflects that implementation science as applied to nutrition has drawn concepts from multiple disciplines, and this lack of conceptual cohesion contributes to the slow progress of the field despite substantial interest. We have identified 2 primary challenges to the field of implementation science arising from this lack of conceptual cohesion.

First, much growth has occurred in the past 5 y in exchange of information about implementation science in nutrition. Nevertheless, the lack of conceptual cohesion has resulted in limited use of existing or creation of new organized venues and media through which to identify scientific goals that would enable clear articulation of options and directions for the field, as well as a base from which to advocate for funding and solicit participation.

Second, the lack of conceptual cohesion has contributed to a tendency to try to understand implementation science in terms of the methods used, or as a compilation of methods and tools, rather than in terms of the questions it asks, the issues it raises, and the contributions it seeks to make. Several prominent definitions of implementation science are focused on methods (20, 29, 31, 32, 36), which explicitly binds implementation science to the study of methods and contributes to the tendency to understand implementation science as a set of methods. Focusing primarily on methods, as in “the study of methods to promote … uptake into [routine clinical] practice” (29), has the further consequences of making the definition convoluted while appearing to minimize other important aspects of implementation, such as the processes related to uptake among target populations. Instead, doing implementation science requires articulating a scientific question (37).

## Defining Implementation Science

The primary challenge that we address is the lack of a straightforward way to describe what implementation science is, which has resulted in confusing what implementation science is with how one goes about doing it. We therefore think it is important to offer a simple definition that articulates the core function of implementation science.

Implementation is the process of activating or making effective actions intended to improve outcomes, and a science is a body of systematized knowledge about a topic. Therefore, implementation science is a body of systematized knowledge about how to improve implementation. This definition rests on 3 principles:

1. Implementation science is distinguished by its aims to learn about the process of implementation.
2. Methods derive from and fit with the aims.
3. Implementation science is built with tacit (as well as expert) knowledge and experiential learning.

### Principle 1: Implementation science is distinguished by its aim to learn about implementation

Implementation science focuses on questions that are broadly about satisfying needs to learn about implementation. Implementation science questions are not limited to making judgments about the worth or value of specific programs or policies (although such questions can be included in an assemblage of implementation science questions), which is the focus of evaluation. Furthermore, implementation science is not tied to the internal logic—the underlying assumptions, program impact paths, etc.—of a specific program or policy. This principle emphasizes the need to ask a broad set of questions that will generate learning about implementation.

As a science, implementation science must ask questions and create knowledge from both discovery and mission orientations. A discovery orientation seeks to create a reservoir of knowledge that can then be applied in situations or to problems, whereas a mission orientation seeks to create knowledge to help accomplish a specific objective (38). These orientations are distinct but not mutually exclusive. Implementation science questions should be inspired by their usefulness, but not necessarily limited to ones that have immediate use. Some examples of questions are:

- How can programming be integrated into and strengthened in existing systems and platforms at national and subnational levels?
- How can data and implementation learning be used to improve quality and coverage of services, equity in who gets to access those services and why, and who is accountable if they cannot get access (39)?
- What conditions, strategies, and methods are needed to enable country-level scale-up of effective interventions?
- How does one motivate individuals to adopt and sustain behavior change?
- How can the capacity, capabilities, motivation, and performance of frontline workers be improved?
- How can programs be sustained at community, program, and institutional levels?
- How can lessons learned in one country be used to improve implementation in other countries in the same region?

### Principle 2: Methods derive from and fit with the aims

As with any science, in implementation science the methods must be selected to match the aims or questions, drawing on theories, frameworks, and methods from a wide array of fields: “… it makes little sense to talk in terms of a set of implementation research methods … it is the question that determines the method used, rather than the method that determines the kinds of questions asked” (33). Articulating first the aims or questions is particularly important for implementation science given its tendency to narrowly focus on methods, as discussed earlier. Aims and questions can be addressed through a combination of methods, including in-depth qualitative methods, which are important to provide richness from the perspectives of actors involved in implementation. Given that implementation is complex and learning needs are complex, mixed methods should be used to encircle an issue. In-depth qualitative methods are well suited for the “how” and “why” questions and to capture tacit knowledge and foster experiential learning. Quantitative methods can answer questions about the extent and distribution of an issue and to what it is related. For example (Box 1), mixed methods, primarily interviews and observations, were used to study the processes of, and influencers on, implementation of an intervention intended to improve infant and young child feeding in Bangladesh (40). Quantitative methods were then used to examine *1*) whether and how various intervention design elements (e.g., training, supervision, mass media) affected the performance of frontline workers in delivering services (41), and *2*) the role of social networks, information diffusion, and social norms in translating services into practice among mothers (42).

**Box 1: Implementation science in Alive & Thrive in Bangladesh**

**Table:** 

Alive & Thrive from late 2008 to 2014 aimed to improve infant and young child feeding in Bangladesh (and 2 other countries) by learning through doing how to design and implement large-scale social behavior change communication intervention. Frontline workers and health volunteers in BRAC, a large non-governmental organization operating throughout the country, provided counseling on infant and young child feeding through home visits. In addition, community mobilization, mass media, and policy advocacy provided messages on various aspects of feeding aimed at national and community leaders, journalists, mothers, family members, health workers, local doctors, and others. A series of implementation science studies were done using a mix of methods to understand how the implementation unfolded in practice; identify bottlenecks; learn about whether and how intervention design elements (e.g., training, supervision, mass media) affected the performance of frontline workers in delivering services; and learn how messages were translated into practice by mothers through social networks, information diffusion, and formation of social norms. The methods used were tailored to each question about implementation and chosen to maximize gaining tacit knowledge and learning from experiences of frontline workers, volunteers, mothers, and others involved in implementation. The questions asked and methods used included:	
**Questions asked**	**Methods used**
How did implementation occur and why?	Development of program impact path diagram; review of training materials; assessment of knowledge of frontline workers and volunteers; structured and semistructured interviews with and observations of frontline workers, volunteers, and mothers; shadowing of frontline workers and volunteers (40)
How did various intervention design elements affect the performance of frontline workers in delivering services?	Survey questionnaires with frontline workers, volunteers, and mothers (41)
What role did social networks, information diffusion, and social norms have in translating services into practice among mothers?	Survey questionnaires with mothers (42)

### Principle 3: Implementation science is built with tacit knowledge and experiential learning

Because implementation necessarily involves multiple entities, collaboration is essential to implementation science. Conducting implementation science requires experts but also requires practitioners who are doing the work of implementation. Therefore, in addition to expert knowledge, tacit knowledge and experiential learning is essential in building implementation science (43). Gaining tacit knowledge and experiential learning requires engaging collaboratively with multiple stakeholder groups (e.g., practitioners, policy makers, researchers, and communities). Such collaboration can articulate priorities, generate aims and questions, identify data sources and methods to answer questions, and determine use of results.

Collaboration among stakeholders means that a shared space must be created. But what are the terms of engagement in this shared space? Who identifies priorities for learning needs? Who articulates the questions? What are the data? What are the methods? Who does the work? Who is the audience? How does it get used? These questions are among many that the development of implementation science will answer.

## Building Implementation Science in Nutrition

Building implementation science in nutrition will be enhanced in 2 ways, by *1*) attending to what is needed for the field of nutrition, and *2*) taking concrete actions to build this science for nutrition. Some examples of what is needed for the field of nutrition are:

- Refocus on studying the implementation of interventions, rather than intervention impacts of implementation. This distinction reflects the difference between implementation science and evaluation science. There is currently an overreliance on studying implementation only as a part of the path to impact in evaluation or in underpowered process evaluations. More studies are needed with outcomes of implementation processes as main outcomes, including feasibility, adoption, acceptance, quality, equity, efficiency, scale, and sustainability.
- Leverage the tacit knowledge of program implementers to consider all aspects of implementation. There is currently an overreliance on studying implementation nested as part of an impact evaluation.
- Study the drivers and processes that affect implementation quality across multiple domains and how to improve it.

Some concrete actions to building implementation science in nutrition are:

- Develop a shared understanding of what implementation science is, what it is not, and how it can be used to improve delivery of interventions, programs, and polices.
- Shift perception of implementation science by stakeholders, including academic institutions, journals, donors, and implementing organizations, in order to build the credibility, acceptance, and importance of implementation as a legitimate and valued science.
- Build capacity to conduct implementation science through multipronged efforts that include formal academic degree training, nondegree short courses, webinars, and other avenues.
- Develop and socialize an implementation science agenda focused on knowledge gaps around the delivery of nutrition interventions, programs, and policies that can be filled through a rigorous study of implementation processes, contexts, and domains.
- Bring program implementers into the implementation science tent by institutionalizing implementation science as a core component of the implementation process among implementing organizations.
- Expand channels of dissemination for implementation science experiences through traditional academic (e.g., peer-reviewed academic journals, academic conference presentations) and nonacademic (i.e., webinars, blogs, web repositories for and email lists to practice communities) avenues.
- Expand funding opportunities for studies of implementation including opportunities that are not linked to larger-scale impact evaluations.

## Conclusion

Whereas evaluation is intended to address questions and render judgments about the worth or value of a program or policy and performance in accordance with its own change theory or internal logic, implementation science is intended to improve implementation. Implementation studies might adopt similar methods and reporting formats and require similar collaborative relations to conduct as evaluation, but the aims and questions are different. What implementation science offers the field of nutrition, as a system of scientific inquiry distinct from evaluation, is its freedom to pursue “big” questions, that is, questions that are not driven by a program's internal logic, including those concerning phenomena on the periphery relevant to program implementation or uptake.

No less significant, conducting research or other forms of assessment that do not easily fit within the bounds of traditional evaluation under the auspices of implementation science helps to create a shared space in which to foreground the learnings derived from these types of studies, which have previously been obscured or failed to find an outlet for dissemination. That is, implementation studies in nutrition, in defying specific designations and/or disciplinary homes, for example, “not policy science, not nutrition, not anthropology, not evaluation,” have historically lacked opportunities for dissemination and discussion within the wider community of practice.

Implementation science aims to generate the learning needed to improve implementation through facilitating collaboration among stakeholders to articulate and pursue the aims; capturing and using tacit knowledge and experiential learning from stakeholders, systems, providers, and recipients; and applying a mix of methods suited to the aims. This definition, and the principles that underly it, provide a simple way to help those who already do, or want to do, implementation science understand and communicate how implementation science is unique and the value that it adds to the current landscape of nutrition priorities, innovations, and the attendant complex learning needs that follow. As with any other science, implementation science encompasses both discovery- and mission-oriented research and centers implementation as the object of study for the purposes of broad-based learning.

## Notes

The authors reported no funding received for this study.

Author disclosures: The authors report no conflicts of interest.

AMW and EAF are members of the board of directors of the Society for Implementation Science in Nutrition. The views expressed are those of the authors and should not be attributed to the Society or its board of directors.
